# Procedural Risks of Carotid Intervention in 19,000 Patients

**DOI:** 10.1016/j.avsg.2020.06.030

**Published:** 2021-01

**Authors:** Kamran A. Gaba, Alison Halliday, Richard Bulbulia, Prem Chana

**Affiliations:** 1 Medical Research Council Population Health Research Unit, Nuffield Department of Population Health, University of Oxford, Oxford, UK; 2 Clinical Trial Service Unit and Epidemiological Studies Unit, Nuffield Department of Population Health, University of Oxford, Oxford, UK; 3 Nuffield Department of Surgical Sciences, University of Oxford, Oxford, UK; 4 Department of Academic Surgery, St Mary's Hospital, Imperial College, London, UK

## Abstract

**Background:**

Randomized controlled trials (RCTs) show that carotid endarterectomy (CEA) and carotid stenting (CAS) reduce long-term stroke risk in symptomatic and asymptomatic patients with carotid artery stenosis. Historical RCTs may not represent contemporary practice and administrative datasets may estimate procedural risks more reliably. We studied procedural risks after carotid intervention in a novel, international administrative data set of 18,997 patients admitted to 28 hospitals across 7 countries.

**Methods:**

Symptomatic and asymptomatic patients undergoing CEA (*n* = 16,220) and CAS (*n* = 2,777) between 2011 and 2015 were studied retrospectively. The primary outcome was in-hospital death within seven days. The secondary outcome was the proportion of patients whose length of hospital stay (LOS) exceeded 2 days. We also describe the rate of computerized tomography brain imaging within 2 days of CEA and CAS (proxy for stroke) as procedural strokes were not reliably recorded.

**Results:**

In symptomatic patients after CEA, mortality was 0.2% [5/2,118] (95% confidence interval: 0.1–0.5), and 57.0% [628/1,101] (54.1–60.0) had prolonged LOS. In asymptomatic patients after CEA, mortality was 0.1% [21/14,102] (0.1–0.2), and 28.5% [2,864/10,039] (27.7–29.4) had prolonged LOS. In symptomatic patients after CAS, mortality was 3.3% [10/307] (1.3–5.2), and 64.3% [144/224] (58.0–70.5) had prolonged LOS. In asymptomatic patients after CAS, mortality was 0.7% [18/2,470] (0.4–1.1), and 27.5% [601/2,187] (25.6–29.4) had prolonged LOS. After CEA, 8.1% [89/1,101] (6.5–9.7) symptomatic patients and 2.1% [207/10,039] (1.8–2.3) asymptomatic patients underwent brain imaging. After CAS, 7.1% [16/224] (4.0–10.7) symptomatic patients and 3.2% [71/2,187] (2.5–4.0) asymptomatic patients underwent brain imaging.

**Conclusions:**

Death and LOS after CEA and CAS were higher in symptomatic than asymptomatic patients. Symptomatic patients undergoing CAS had particularly increased risk of death. This may be partly explained by case selection, with more comorbid patients preferentially undergoing CAS. While RCTs effectively compare long-term efficacy of CEA versus CAS, administrative datasets can provide reliable estimates of contemporary procedural risks.

## Highlights

• We studied risks after carotid intervention in an administrative data set.
• Carotid intervention outcomes were worse in symptomatic than asymptomatic patients.
• Symptomatic patients undergoing carotid stenting had increased mortality risk.
• Administrative datasets provide reliable estimates of contemporary procedural risks.

## Introduction

Randomized controlled trials (RCTs) are the “gold standard” in comparing the effectiveness of a new intervention (medical or surgical) to established practice. When rigorously conducted, they have several advantages. Random allocation of individuals to either the “intervention” or “control” group eliminates selection bias and maximizes the possibility that the 2 groups will be identical in demographics, comorbidity, and any other potentially confounding factors. Double-blinding ensures that there is no difference in treatment of either group of individuals and also minimizes the one-way effect of placebo. Intention-to-treat analysis also minimizes the effect of treatment switching or withdrawal on outcomes, whereas meta-analyses of RCTs remain the highest form of evidence and inform clinical practice. However, RCTs are expensive, labour-intensive and require time-consuming data entry and follow-up at several collaborating centers.

RCTs have shown that carotid endarterectomy (CEA) and carotid stenting (CAS) reduce long-term stroke risk in highly selected symptomatic and asymptomatic patients with CAS, compared with best medical therapy (BMT). Procedural outcomes in RCTs show an increased stroke risk after CAS (compared with CEA), especially in symptomatic patients, but the risk of myocardial infarction is higher after CEA than CAS.^1^, ^2^, ^3^, ^4^, ^5^, ^6^, ^7^, ^8^ However, this may not be generalizable to contemporary practice as BMT and experience with surgical techniques and endovascular devices have improved since the first CAS trial started recruiting patients over 20 years ago. Centers and surgeons/interventionists (and their patients) enrolled in RCTs may also differ from the general population while RCTs comparing CEA versus CAS had varying use of embolic protection devices. One RCT^4^ ignored strokes lasting less than 7 days, confounding results, while another^9^ only included patients at high operative risk. Therefore, it is important to describe contemporary outcomes after CEA and CAS.

### The Dr Foster Global Comparators’ (GC) Project

Large administrative datasets may more accurately reflect the contemporary effect of advances in modern surgical practice and BMT on procedural outcomes after CEA and CAS than results derived from historical RCTs. They usually contain more patients than RCTs and capture more events. The population included in registries will likely also include patients who were ineligible for inclusion in RCTs and therefore may be more representative of the general population.

We had access to a large administrative data set, established as part of the Dr Foster GC Project. This is an international, benchmarking, collaborating initiative that collects and shares data from academic institutions around the world to allow quality improvement and the identification of best practice, thereby improving outcomes in patient care. This has been demonstrated in both elective colorectal surgery^10^ and emergency general surgery.^11^ It is a clinician-led, not-for-profit subsidiary of Telstra Corporation.

There are 41 collaborating centers, across 4 continents, but the database is managed by a team of analysts based in London. They liaise closely with the contributing centers and receive quarterly data updates.

### Aim

The aim of this study was to assess contemporary procedural risks in 18,997 symptomatic and asymptomatic patients who underwent CEA or CAS in 28 hospitals across 7 countries.

## Materials and Methods

### Participants

Data relating to all patients who underwent CEA or CAS between 2011 and 2015 were obtained retrospectively from 28 centers participating in GC from Australia, Belgium, Denmark, Finland, England, the Netherlands, and the United States of America. For English hospitals, further data were obtained from the Hospital Episodes Statistics database. For other countries, electronic inpatient records were used.

Data relating to age, demographics, primary diagnosis, and comorbidities (namely diabetes mellitus, obesity, and heart disease) were collected. Patients were defined as symptomatic if they had suffered a stroke, transient ischemic attack (TIA), or amaurosis fugax in the 6 months before their CEA or CAS. If they had not, they were deemed asymptomatic. A stroke was diagnosed if a focal neurological impairment (of ischemic etiology) lasted more than 24 hours. If the neurological impairment resolved within 24 hours, a TIA was diagnosed.

All participating centers provided consent for use of their data for research. No identifiable patient data were used, so consent from individuals was not required. Centers were excluded if their data set was incomplete. Any admissions not related to CEA or CAS were excluded.

### Outcomes

The primary outcome was 7-day in-hospital death after CEA and CAS. Deaths outside hospital or after 7 days postprocedure were not captured.

The secondary outcome was the proportion of patients with a postprocedural length of hospital stay (LOS) greater than 2 days as a proxy measure for suspected or actual complications.

Difficulties arose in accurately determining procedural strokes due to the method of coding being unable to differentiate between patients who had suffered strokes before or after CEA or CAS (especially if symptomatic). Therefore, an exploratory analysis was performed where computerized tomography (CT) scan of the head within 2 days of CEA or CAS was used as a surrogate measure for suspected or actual procedural stroke.

### Statistics

Using the International Classification of Diseases-9 and -10 codes for CEA and CAS, data from participating countries were collected and analyzed using the statistical software package R. 95% confidence intervals were calculated for all outcome measures. Chi-squared and Mann-Whitney *U*-tests were used to compare CEA and CAS cohort characteristics. A *P*-value ≤0.05 was deemed statistically significant.

## Results

A total of 16,220 CEA and 2,777 CAS procedures were included in this study. American centers (*n* = 9) recruited 8,363 patients (44.0%), Australian centers (*n* = 4) recruited 1,742 patients (9.2%), and European centers (*n* = 15) recruited 8,892 patients (46.8%).

### Demographics and Comorbidities

The median age of CEA patients was 71 (interquartile range: 64–77) years and 69 (interquartile range: 61–76) years for CAS (Table I). The proportion of women was similar between CEA and CAS groups.

**Table I:** Characteristics of patients undergoing carotid intervention in the GC data set. Abbreviation–IQR, interquartile range

Characteristics	CEA	CAS	*P*-value
Procedural data			
Procedures (*n*)	16,220	2,777	
Hospitals (*n*)	28	23	
Demographics			
Median age (IQR)	71.0 (64–77)	69.0 (61–76)	0.40
Percentage Female	34.4	36.0	0.77
Primary diagnosis (%)			
Symptomatic	15.9	12.2	0.41
Ischemic stroke	13.1	11.1	0.66
TIA	2.8	1.1	0.31
Comorbidity (%)			
Diabetes mellitus	23.5	28.2	0.52
Obesity	5.6	7.0	0.77
IHD	28.4	44.7	***0.01***

Bold indicates statistically significant of *P* ≤ 0.05.

Asymptomatic patients accounted for 84.6% [16,079/18,997] of the total cohort. Ischemic stroke was the most common indication for CEA or CAS in symptomatic patients, followed by TIA (Table I). Patients undergoing CEA and CAS had similar rates of diabetes mellitus and obesity. Ischemic heart disease was significantly more common in patients who underwent CAS (Table I).

### Outcomes

After CEA, symptomatic patients had similar mortality rates but longer LOS than asymptomatic patients (Table II). After CAS, symptomatic patients had a higher mortality and LOS than asymptomatic patients (Table II).

**Table II:** Procedural outcomes (with 95% confidence intervals) after carotid intervention

Rates (95% CI)	Symptomatic		Asymptomatic	
	CEA	CAS	CEA	CAS
7-day in-hospital mortality	0.2% [5/2,118] (0.1–0.5)	3.3% [10/307] (1.3–5.2)	0.1% [21/14,102] (0.1–0.2)	0.7% [18/2,470] (0.4–1.1)
LOS more than 2 days	57.0% [628/1,101] (54.1–60.0)	64.3% [144/224] (58.0–70.5)	28.5% [2,864/10,039] (27.7–29.4)	27.5% [601/2,187] (25.6–29.4)
CT head within 2 days of procedure	8.1% [89/1,101] (6.5–9.7)	7.1% [16/224] (4.0–10.7)	2.1% [207/10,039] (1.8–2.3)	3.2% [71/2,187] (2.5–4.0)

CI, confidence interval.

The rate of CT head within 2 days of the procedure was higher for symptomatic than asymptomatic patients for both CEA and CAS (Table II).

## Discussion

After CEA and CAS, we found that death and prolonged LOS were numerically higher in symptomatic than asymptomatic patients. Death rates were numerically highest in symptomatic patients undergoing CAS. This may be due to case selection, with more comorbid patients undergoing CAS. Our results also suggest that symptomatic patients may be at a greater risk of procedural stroke after both CEA and CAS than asymptomatic patients. As administrative datasets may reflect advances in surgical practice/BMT and “real-world” practice (including active patient selection and experience of interventionists) more accurately than RCTs, these findings provide reliable estimates of procedural rates of death, LOS, and brain imaging after CEA and CAS, in a relatively large sample of contemporary practice.

### CEA Outcomes

Mortality after CEA was broadly consistent with findings from RCTs (Table III). The Carotid Revascularization Endarterectomy versus Stenting (CREST)-1 trial^1^^,^^2^ reported no deaths after CEA in their small subgroup of symptomatic patients while the endarterectomy versus angioplasty in patients with Symptomatic Severe Carotid Stenosis (EVA-3S),^3^ Carotid and Vertebral Artery Transluminal Angioplasty Study (CAVATAS),^4^ Stent-Protected Angioplasty versus Carotid Endarterectomy (SPACE)-1^5^ and International Carotid Stenting Study (ICSS)^6^ trials reported higher 30-day mortality rates than our study. This may be due to temporal improvements in outcomes, with increased experience, more effective BMT, technological advancement, and better patient selection.

**Table III:** Comparison of procedural mortality between the GC data set and RCTs

Mortality rate	CEA	CAS
Symptomatic patients		
GC data	**0.2% [5/2,118]**	**3.3% [10/307]**
EVA-3S	1.2% [3/259]	0.8% [2/261]
SPACE-1	0.9% [5/584]	0.7% [4/599]
ICSS	0.5% [4/821]	1.3% [11/828]
CAVATAS	1.6% [4/253]	2.8% [7/251]
CREST-1	0.0% [0/648]	0.4% [3/673]
Asymptomatic patients		
GC data	**0.1% [21/14,102]**	**0.7% [18/2,470]**
CREST-1	0.0% [0/581]	0.0% [0/600]
ACT-1	0.3% [1/348]	0.1% [1/1,072]
SPACE-2	0.0% [0/203]	0.0% [0/197]

Bold indicates the data compared to RCT findings.

Mortality after CEA in asymptomatic patients was broadly consistent with the Asymptomatic Carotid Trial (ACT)-1 RCT.^7^ While CREST-1^2^ and SPACE-2^8^ reported no procedural deaths after CEA in asymptomatic patients, their cohorts were small.

### CAS Outcomes

Mortality after CAS in symptomatic patients was similar to CAVATAS^4^ but higher than CREST-1^2^, EVA-3S^3^, SPACE-1^5^_,_ and ICSS (Table III).^6^ This might be explained by case selection, with more comorbid patients preferentially undergoing CAS (ischemic heart disease was significantly more common in CAS than CEA patients). Our substantially elevated mortality after CAS in symptomatic patients possibly reflects the influence of active patient selection on procedural outcomes (eliminated by randomization in RCTs) or the smaller number of symptomatic patients undergoing CAS in our data set. Nevertheless, this trend for excess mortality after CAS in symptomatic patients is consistent with RCT findings and supported by a systematic review that reported procedural risks of stroke/death after CAS exceeding 10% in 28% of reports from registries in symptomatic patients.^12^

Mortality after CAS in asymptomatic patients was broadly consistent with findings from ACT-1 (Table III).^7^ While CREST-1^2^ and SPACE-2^8^ reported no deaths after CAS in asymptomatic patients, the results should be cautiously interpreted due to small patient cohorts.

### Proxy Measures

As administrative data sets may not be primarily designed for research purposes, they may not contain all data that may be useful for researchers. For example, the GC data set did not include comorbidities such as hypertension or chronic kidney disease. There were also difficulties in accurately identifying procedural complications (such as new strokes after CEA or CAS, especially in symptomatic patients). We therefore used surrogate markers as proxies to make inferences regarding rates of complications after CEA and CAS.

Prolonged LOS is rarely used as an outcome measure. In an American Registry study, McPhee et al.^13^ reported a longer LOS for symptomatic patients undergoing CEA and CAS.^13^ For asymptomatic patients, they reported similar LOS after CEA and CAS.^13^ This is broadly consistent with our findings (Table II) and suggests that prolonged LOS may be a useful, proxy outcome measure for suspected or actual procedural complications after CEA and CAS.

To date, no study has used the rate of CT brain imaging within 2 days of CEA or CAS as a proxy for suspected procedural stroke. Our reported CT head scan rate after CEA in asymptomatic patients (2.1%) was consistent with 30-day procedural stroke rates reported in CREST-1^2^ (1.4%), ACT-1^7^ (1.4%), and SPACE-2^8^ (2.0%). Rates of CT head scan after CAS in symptomatic patients (7.1%) were also consistent with 30-day procedural stroke rates reported by CREST-1^2^ (5.5%), EVA-3S^3^ (8.8%), CAVATAS^4^ (7.2%), SPACE-1^5^ (7.5%), and ICSS^6^ (7.0%). Rates of CT head scan after CAS in asymptomatic patients (3.2%) were consistent with 30-day procedural stroke rates reported in CREST-1^2^ (2.5%), ACT-1^7^ (2.8%), and SPACE-2^8^ (2.5%). Rates of CT head scan after CEA in symptomatic patients (8.1%) were also consistent with 30-day procedural stroke rates reported in CAVATAS^4^ (8.3%) and SPACE-1^5^ (6.2%) but substantially higher than CREST-1^2^ (3.2%), EVA-3S^3^ (2.7%), and ICSS^6^ (3.3%). Taken together, our findings suggest that our surrogate outcome measure is a relatively sensitive measure of procedural stroke as it broadly supports RCT findings. However, it may overestimate post-CEA stroke rates in symptomatic patients, possibly due to the relatively smaller number of symptomatic patients in this dataset. Further refinement may be needed to improve its sensitivity and specificity before this outcome measure can be used more widely.

### Limitations

This study has several limitations: first, GC centers were technologically advanced, academic, and interested in quality improvement, so their practice may not be generalizable; second, there may be selection bias with more comorbid patients undergoing CAS; third, deaths outside hospital or after 7 days postprocedure were not captured; finally, our proxy measure may overestimate procedural strokes in symptomatic patients undergoing CEA possibly due to the relatively smaller number of symptomatic patients in this data set.

## Conclusions

Death and prolonged LOS were numerically higher in symptomatic than asymptomatic patients undergoing CEA and CAS. Procedural death was numerically highest in symptomatic patients undergoing CAS. This may be due to case selection, with more comorbid patients undergoing CAS. As administrative datasets may more accurately reflect contemporary practice than RCTs, these findings provide reliable estimates of current absolute procedural risks after CEA and CAS.
